# MEK1 is associated with carboplatin resistance and is a prognostic biomarker in epithelial ovarian cancer

**DOI:** 10.1186/1471-2407-14-837

**Published:** 2014-11-18

**Authors:** Zsófia Pénzváltó, András Lánczky, Julianna Lénárt, Nóra Meggyesházi, Tibor Krenács, Norbert Szoboszlai, Carsten Denkert, Imre Pete, Balázs Győrffy

**Affiliations:** MTA-TTK Lendület Cancer Biomarker Research Group, Budapest, Hungary; 1st Department of Pathology and Experimental Cancer Research, Budapest, Hungary; MTA-SE Tumor Progression Group, Budapest, Hungary; Eötvös Loránd University, Institute of Chemistry, Budapest, Hungary; Institut of Pathology, Charité Universitatsmedizin, Berlin, Germany; National Institute of Oncology, Budapest, Hungary; MTA-SE Pediatrics and Nephrology Research Group, Budapest, Hungary; 2nd Department of Pediatrics, Semmelweis University, Budapest, Hungary

**Keywords:** Ovarian cancer, Chemotherapy, Carboplatin, MEK

## Abstract

**Background:**

Primary systemic treatment for ovarian cancer is surgery, followed by platinum based chemotherapy. Platinum resistant cancers progress/recur in approximately 25% of cases within six months. We aimed to identify clinically useful biomarkers of platinum resistance.

**Methods:**

A database of ovarian cancer transcriptomic datasets including treatment and response information was set up by mining the GEO and TCGA repositories. Receiver operator characteristics (ROC) analysis was performed in R for each gene and these were then ranked using their achieved area under the curve (AUC) values. The most significant candidates were selected and *in vitro* functionally evaluated in four epithelial ovarian cancer cell lines (SKOV-3-, CAOV-3, ES-2 and OVCAR-3), using gene silencing combined with drug treatment in viability and apoptosis assays. We collected 94 tumor samples and the strongest candidate was validated by IHC and qRT-PCR in these.

**Results:**

All together 1,452 eligible patients were identified. Based on the ROC analysis the eight most significant genes were JRK, CNOT8, RTF1, CCT3, NFAT2CIP, MEK1, FUBP1 and CSDE1. Silencing of MEK1, CSDE1, CNOT8 and RTF1, and pharmacological inhibition of MEK1 caused significant sensitization in the cell lines. Of the eight genes, *JRK* (p = 3.2E-05), *MEK1* (p = 0.0078), *FUBP1* (p = 0.014) and *CNOT8* (p = 0.00022) also correlated to progression free survival. The correlation between the best biomarker candidate *MEK1* and survival was validated in two independent cohorts by qRT-PCR (n = 34, HR = 5.8, p = 0.003) and IHC (n = 59, HR = 4.3, p = 0.033).

**Conclusion:**

We identified *MEK1* as a promising prognostic biomarker candidate correlated to response to platinum based chemotherapy in ovarian cancer.

**Electronic supplementary material:**

The online version of this article (doi:10.1186/1471-2407-14-837) contains supplementary material, which is available to authorized users.

## Background

Ovarian cancer is the fifth leading cause of cancer death among women in the USA, with approximately 22,000 new cases and 14,000 deaths per year [1]. Primary treatment includes surgery and platinum-based chemotherapy. To date, with the exception of bevacizumab, no successful trial has been conducted identifying any efficient targeted therapy for ovarian cancer patients [2, 3]. Thus, the platinum-taxane chemotherapy still represents the gold standard of treatment. Following chemotherapy, platinum-resistant cancer recurs (or progresses despite the therapy) in approximately 25% of patients within six months [4] and the overall 5-year survival is only 30% [5].

Platinum agents bind DNA forming inter- and intra-strand DNA adducts [6]. Cellular perception of these DNA adducts leads to the activation of DNA-damage mediated apoptotic pathways. Resistance against carboplatin can evolve by three principal mechanisms: reduction of intracellular drug concentration (involving alterations in CTR1, CTR2, ATP7B, GST), changes in DNA repair (ERCC1, MLH1, MSH2, BRCA1/2) or modification of cellular response (TP53, ERBB2, CCNE) which mechanisms have been discussed previously [7, 8].

Although many single genes are well-known to be involved in the biological machinery of resistance against platinum agents, no approved predictive biomarker is yet available. In addition, some array based studies promised higher prognostic and predictive efficiency [9]. A 14-gene predictive model (based on specimens from 79 patients) was capable to discriminate women at risk for early versus late relapse after initial chemotherapy [10]. Spentzos and colleagues identified a 115-gene expression set as a prognostic marker (Ovarian Cancer Prognostic Profile) in 68 patients [11]. A 300-gene ovarian prognostic index was identified in 80 patients and validated in an independent set of 118 patients [12]. However, these gene sets share only a minimal number of genes, which draws attention to the following important points: high sample numbers are necessary to have a representative picture of the patient population, identical platforms must be used, and unbiased pre-processing methods have to be applied [13].

In present study, our aim was to identify predictive gene expression markers based on a large patient cohort established using reproducible analysis steps. The *in silico* identified strongest gene candidates were then further assessed *in vitro*. Finally, clinical applicability of the most promising candidate was tested in two independent patient cohorts.

## Methods

### Set-up of microarray databank

We searched GEO (http://www.pubmed.com/geo) and TCGA (http://cancergenome.nih.gov) to identify datasets suitable for the analysis. In this, the keywords “ovarian”, “cancer”, “survival”, “GPL96”, “GPL570” and “GPL571” were used. Only publications with available raw microarray gene expression data, clinical treatment and response information, and at least 20 patients were included. Only three microarray platforms, GPL96 (Affymetrix HG-U133A), GPL570 (Affymetrix HG-U133 Plus 2.0), and GPL571/GPL3921 (Affymetrix HG-U133A 2.0) were considered.

### Bioinformatic processing

**Figure 1:**
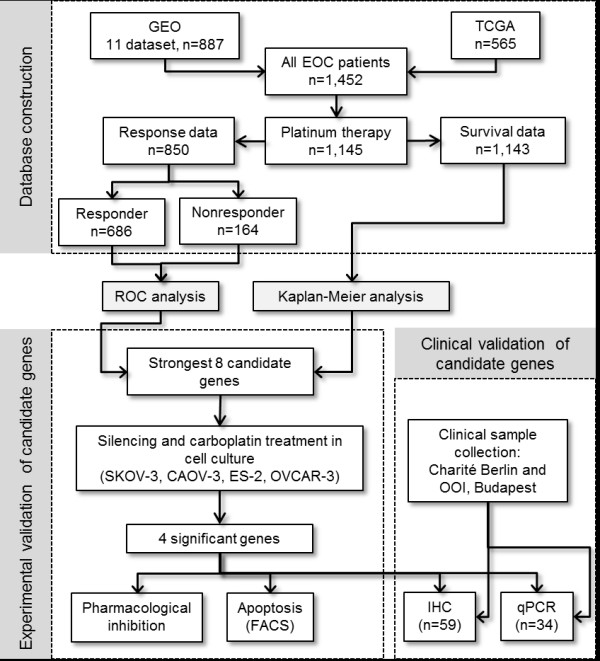
**Overview of the study.**

Raw .CEL files were MAS5 normalized in the R statistical environment (http://www.r-project.org) using the affy Bioconductor library [14]. For the analysis, only probes measured on GPL96, GPL570 and GPL571/GPL3921 were retained (n = 22,277). Then, a second scaling normalization was performed to set the average expression on each chip to 1000 to reduce batch effects [15]. The package “roc” was used to calculate AUC and significance, and to plot ROC curves to compare responders and non-responders. Kaplan-Meier survival plots were calculated and plotted in R using the “survplot” function of the “survival” Bioconductor package to assess the correlation between survival and gene expression [16]. To elaborate the three previously reported potential mediator mechanism of MEK1 in carboplatin resistance *(seeDiscussion),* we have set up metagenes using the mean expression of genes involved in the AKT pathway (AKT1, PI3KCA, MDM2, MTOR) and epithelial–mesenchymal transition inducers (EMT; including CDH1, SNAI1, SNAI2, ZEB1, ZEB2, E47, KLF8, TWIST, TCF4, SIX1, FOXC2). Finally, Spearman rank correlation was computed between expression of MEK1 and ERCC1, and the AKT and EMT metagenes. An overview of the study and the bioinformatical processing is exhibited in Figure 1*.*

### Cell culture

The epithelial ovarian cancer cell lines (obtained from ATCC) SKOV-3, CAOV-3, ES-2 and OVCAR-3 were cultured in RPMI 1640 media with 10% FBS and antibiotics (penicillin-streptomycin, amphotericin B and tetracycline). Mycoplasma tests using Mycosensor PCR Assay Kit (Agilent) were performed before starting the experiments and BM-Cyclin (Agilent) or ciprofloxacin was used to eliminate contamination.

### Authentication of the cell lines

Authentication was performed for the investigated cell lines using short tandem repeat (STR) analysis of 10 specific loci in the human genome and a mouse specific marker. DNA was isolated from the cell lines with DNeasy Blood and Tissue Kit (Quiagen), quantity and quality of isolated DNA were measured by Nanodrop ND-1000 system. DNA (A260) and protein (A280) concentrations and sample purity (260/280 ratio) were measured, and only high quality DNA was used for SRT analysis. Authentication was carried out by StemElite ID System at the Fragment Analysis Facility, Johns Hopkins University. STR profiles of the cell lines were compared to the STR profile database of the Leibniz Institute DSMZ - German Collection of Microorganisms and Cell Cultures (http://www.dsmz.de). All four cell lines included in this study were contamination-free.

### Chemosensitivity testing

MTT Cell Proliferation Kit I (Roche) was used to test drug sensitivity of the cell lines. In this, 10,000 cells/well were seeded in 90 μl medium onto 96-well plates in six repeats. After overnight incubation, carboplatin was added in increasing grade of approximately 2 μM to 1 mM (corresponding to 0x-40x of the clinically administered dose) in 10 μl water solution (the table of used concentrations is presented in Additional file 1: Table S1*.*). Control wells were treated with vehicle. After 48 hours of drug treatment, the experiment was terminated and cells were stained. The reaction was quantified by measurement of absorbance at 595 nm. The measured value was corrected with the reference measured at 690 nm. GraphPad Prism 5 was used to determine IC50 values and to visualize results.

### siRNA transfection

We optimized transfection by employing GAPDH positive control siRNA (Silencer Select, Life Technologies). Efficacy of silencing was measured by qRT-PCR (Roche LightCycler 480 system). The highest silencing efficacy was achieved with a siRNA concentration of 30 nM and Lipofectamine RNAiMax transfection reagent. JRK was not expressed in the selected cell lines and was therefore excluded from the silencing experiment. Silencing efficacy of two pre-designed Silencer Select siRNAs per gene were assessed for each selected gene. The oligo with higher silencing efficiency was selected for performing the drug combined silencing experiment. The ID-s of the used siRNA-s are presented in Table 1.

**Table 1:** **Candidate biomarkers**

Gene		Platinum-treated patients (n = 1,152)		siRNA
Affy ID	Gene	ROC (AUC)	ROC (p value)	siRNA ID
**214692_s_at**	**JRK**	0.62	1.34*10^-7^	-
**200910_at**	**CCT3**	0.62	3.50*10^-7^	s14397
**212301_at**	**RTF1**	0.62	5.87*10^-7^	s23185
**202670_at**	**MEK1**	0.61	1.75*10^-6^	s1167
**214094_at**	**FUBP1**	0.61	2.25*10^-6^	s16966
**202162_s_at**	**CNOT8**	0.61	3.07*10^-6^	s17848
**217527_s_at**	**NFATC2IP**	0.61	3.69*10^-6^	s39617
**202646_s_at**	**CSDE1**	0.6	4.18*10^-6^	s15374
**214093_s_at**	**FUBP1**	0.61	5.34*10^-6^	s16966

The eight strongest genes selected for *in vitro* validation including the results of the bioinformatic processing performed using transcriptomic data of 1,145 platinum-treated ovarian cancer patients.

### Combination of gene silencing and drug treatment

To investigate the role of selected genes in carboplatin resistance, we combined gene silencing and drug treatment. In this, 10,000 cells/well were transfected and seeded in 90 μl medium onto 96-well plates in six repeats. After overnight incubation, carboplatin was added to the cells at the IC50 dose for each cell line. After a 48 hour drug treatment, cells were stained by MTT. In each siRNA transfected group, absorbance values of the drug treated group were normalized to the untreated group. T-test was used to analyze the difference between negative control siRNA transfected (carboplatin treated) and target gene siRNA transfected (carboplatin treated) groups. Significance level was set at p < 0.01.

### Apoptosis analysis

Change in the apoptotic ratio of carboplatin treated cells as a result of silencing for each of the five genes was measured by FACS. Measurements were performed in triplicate. After overnight incubation, transfected CAOV-3 cells were treated with the IC50 dose of carboplatin for 48 hours. Then, apoptosis rate was detected by FITC Annexin V Apoptosis Detection Kit I (BD Pharmingen) according to the user’s manual in BD FACS Aria I. Apoptotic ratio in the silenced groups was compared to the negative control siRNA transfected cohort. T-test was used to analyze the difference between groups. Significance level was set at p < 0.05.

### Pharmacologic MEK1 inhibition

As a pilot experiment, PD0325901, a selective MEK1 inhibitor was used to investigate the sensitizing effect of MEK1 inhibition. Two cell lines (SKOV-3, CAOV-3) were treated with increasing concentrations of PD0325901 for 48 hours and then stained with MTT. After determining the sensitivity profile for each cell line against PD0325901, an experiment was set up using the approximate IC50 or a less effective dose of carboplatin, alone and in combination with an effective dose of PD0325901. PD0325901 was dissolved in DMSO, carboplatin was dissolved in water, and DMSO alone was used as a vehicle. Viability was normalized to the vehicle treated control; t-test was used to evaluate the results. Significance level was set at p < 0.05.

### Clinical sample collection

Fresh-frozen and paraffin-embedded samples were collected at the National Institute of Cancer (OOI) Budapest, Hungary as described previously [17] and at the Charité Universitätsmedizin Berlin, Germany between 2005 and 2010. For the qRT-PCR measurements, samples were stored at -80 Celsius degrees until RNA isolation. Tissue microarray samples were constructed at the Pathology Institute of the Charité Medical University Berlin. The institutional ethic committees (Ethikausschuss 1 am Campus Charité Mitte and Országos Onkológiai Intézet, Intézeti Kutatásetikai Bizottság - OOI IKEB), approved the study with following reference numbers: EA1/139/05 Amend 2013 (Charité) and OOI-Ált-9444-1/2013/59 (OOI).

### RNA isolation and quality control

Frozen biopsy samples were lysed and homogenized in the mixture of 300 μl GITC containing lysis buffer and 3 μl b-mercaptoethanol by Polytron homogenizator for 30–40 sec., then digested in Proteinase K solution at 55 Celsius for 10 min. RNeasy kit (Quiagen) was used for RNA isolation. After removing genomic DNA by DNase I treatment, the total RNA was eluted in 50 μl RNase free water. Quantity and quality of the isolated RNA were tested by Nanodrop1000 system and by gel electrophoresis using Agilent Bioanalyzer system. Only samples providing high quality, intact total RNA and showing regular 18S and 28S ribosomal RNA bend pattern on the Bioanalyzer analysis were used for PCR.

### Immunhistochemistry

TMA blocks were cut 4 μm thick sections for immunohistochemistry onto charged SuperFrost Ultra Plus glass slides (Menzel). Routine dewaxing of the sections in xylene and descending ethanol series was followed by endogenous peroxidase blocking using 1% hydrogen peroxide in methanol for 30 min. For antigen retrieval sections were boiled (~100°C) in 500 ml of 0.01 M sodium citrate-citric acid (citrate pH 6.0) for 40 min in a microwave oven. After cooling, sections were treated using a 1% bovine serum albumin sodium azide solution for 20 min. Sections were then sequentially incubated using rabbit anti-MEK1 (1:50; HPA026430, Sigma Aldrich) overnight, then with NovoLink detection kit (Leica-NovoCastra) including the post-primary reagent, and then 20 min with polymer peroxidase detection reagent. Peroxidase activity was revealed using a DAB (diaminobenzidine) hydrogen peroxide chromogen-substrate kit for 3–8 min under microscopic control. Between incubations, the sections were washed using 0.1 M Tris–HCl (pH 7.4) buffered saline (TBS), and finally counterstained with hematoxylin. Immunostained slides were digitalized with a Pannoramic Scan 150 (3DHISTECH) under automated white balance using ×20/NA0.8 Zeiss Plan Apochromat objective and a Hitachi HV-F22 3-chip CCD SXGA camera, then analyzed using the Pannoramic Viewer 1.52.2 software through a 24″ Benq LED monitor. The average intensity from four samples per patients was taken for statistical analysis.

### qRT-PCR measurements

Reverse transcriptions were made with SuperScript II Reverse Transcriptase according to the user’s manual, from all RNA samples which fulfilled the quality criteria. LightCycler 480 DNA SybrGreen Master I (Roche) and LightCycler 480 instrument (Roche) were used for qRT-PCR. Gene specific primers were designed using Primer3 software, GAPDH was used as an internal control. All measurements were performed in triplicate. For the immunohistochemistry and qRT-PCR measurements, Cox survival analysis was done to compare the performance of the candidate genes. Kaplan-Meier survival plots were generated using WinSTAT 2013 for Microsoft Excel (Robert K. Fitch Software). In the survival analysis quartiles were used as cutoff values and the significance threshold was set at p < 0.05.

## Results

### Database construction

We identified 1,452 patients in 8 datasets meeting our criteria in GEO and TCGA (the seven GEO datasets are: GSE3149, GSE14764, GSE9891, GSE15622, GSE19829, GSE26712 and GSE18520). The average follow-up for relapse-free survival is 24.8 months with 731 progressions. Of these patients, 1,145 received a platinum-based chemotherapy and 630 received taxol (614 patients received both taxol and platinum).

### Bioinformatic processing

Using Jetset [18] we have filtered for probe set quality and included only reliable and specific probe sets in the statistical evaluation. ROC analysis was performed for all genes, and the eight genes showing the highest AUC value and highest significance were selected for further experiments. The strongest biomarker candidates are summarized in Table 1. Beside the high AUC values, high expressions of JRK (p = 3.2E-05), CNOT8 (p = 2.2E-04), FUBP1 (p = 0.014) and MEK1 (p = 0.0078) also correlated with worse relapse-free survival.

### Chemosensitivity testing

**Figure 2:**
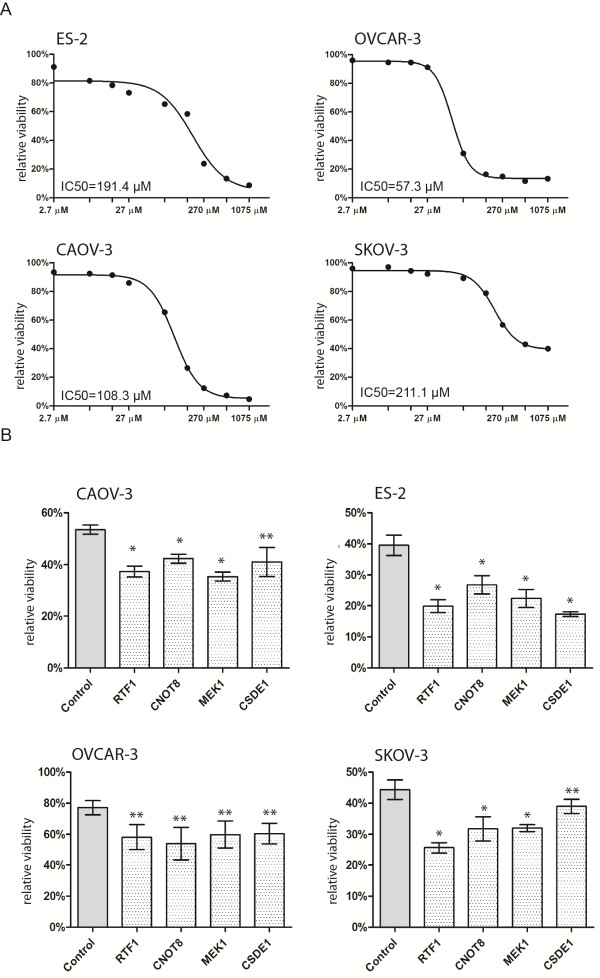
**Carboplatin sensitivity and silencing of the candidate genes.** Dose–response curves of each cell line against carboplatin, after 48 hours drug administration **(A)**. Relative viability after 48 hours carboplatin administration and silencing of four genes compared to the negative control siRNA transfected groups in each of the four cell lines (mean with SEM) *p < 0.001, **p < 0.01 **(B)**.

Sensitivity of the investigated cell lines against carboplatin varied considerably. OVCAR-3 was the most sensitive cell line, with an approximate IC50 of 57.3 μM, SKOV-3 was the most resistant, with an approximate IC50 of 211 μM and the dose–response curve didn’t reach the baseline, even at the highest concentration, corresponding approximately to the 40× of the clinically administered dose. The dose–response curves of the four cell lines are exhibited in Figure 2A*.*

### Combination of gene silencing and carboplatin treatment

The silencing efficacy of the used siRNAs compared to a negative siRNA transfected control (measured by qRT-PCR in triplicates) were 97.7% (CCT3), 98.6% (RTF1), 65% (NFAT2CIP), 98.01% (MEK1), 93.6% (CSDE1), 46.6% (FUBP1), and 99.6% (CNOT8)*.* To observe the role of the selected genes in carboplatin resistance, we combined gene silencing and carboplatin treatment. After 48 hours of treatment, cells were stained with MTT. In each siRNA transfected group, absorbance values of the drug treated group were normalized to the untreated group. As expected, viability of the carboplatin treated cells was 53.6 percent of the viability of the untreated cells, in the negative control siRNA transfected group (in average of the four cell lines). In contrast, in case the target genes were silenced, viability after carboplatin treatment decreased with 5.2% to 26% compared to the negative control siRNA transfected, carboplatin treated group (depending on cell line and gene). Four of the eight investigated genes had significant sensitization effect in all four cell lines, namely RTF1, CSDE1, CNOT8 and MEK1 (p < 0.01). Results of the silencing experiments are exhibited in Figure 2B (non-significant results are not shown).

### Apoptosis assay

**Figure 3:**
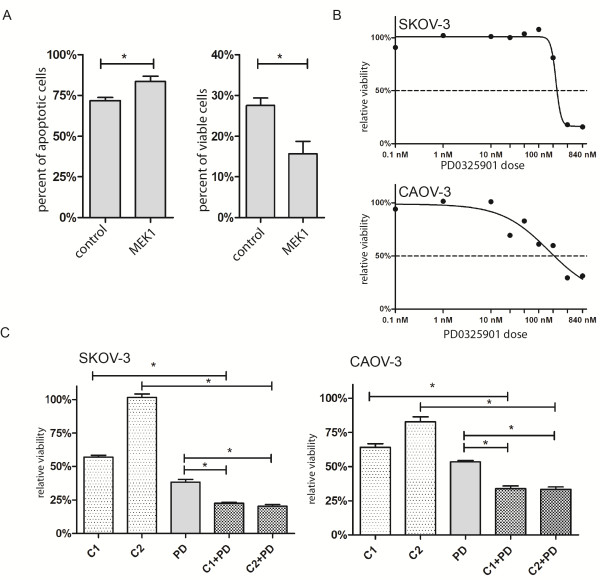
**MEK1 inhibition with carboplatin treatment.** Silencing of MEK1 significantly increases the ratio of the apoptotic cells, and decrease the ratio of the viable cells after 48 hours carboplatin treatment compared to the negative control siRNA transfected cells. *: p < 0.05 **(A)** Dose–response curves of SKOV-3 and CAOV-3 cell lines against the MEK1 inhibitor PD0325901 **(B)**. Effects of 48 hour treatment with carboplatin and PD0325901 as single agents and in combination. SKOV-3: C1: 212 μM carboplatin, C2: 141 μM carboplatin, PD: 554 nM PD0325901 in SKOV3 cell line. CAOV-3: C1: 111 μM carboplatin, C2: 74 μM carboplatin, PD: 277 nM PD0325901 in CAOV-3 cell line (mean with SEM) *p < 0.0001 **C)**.

Silencing of MEK1 in 300,000 cells caused significant increase in the number of apoptotic cells and significant decrease in the number of viable cells after 48 hours of carboplatin treatment (p = 0.0365, Figure 3A). Silencing of the other four genes had no significant effect on the ratio of apoptotic cells *(data not shown)*.

### Pharmacologic MEK1 inhibition

The selective MEK1 inhibitor PD0325901 was effective in both investigated cell lines (SKOV-3 and CAOV-3). SKOV-3 showed higher resistance than CAOV-3 against single agent PD0325901. Combination treatment was performed to detect potential synergistic effect of PD0325901 and carboplatin. The combination treatments had stronger cytotoxic effect compared to monotherapy treatments (p < 0.0001, see Figure 3C). Interestingly, combination of sub-optimal dose of carboplatin with PD0325901 resulted in massive viability decrease (p < 0.0001). The dose–response curves for PD0325901 are exhibited in Figure 3B.

### qRT-PCR measurements

**Figure 4:**
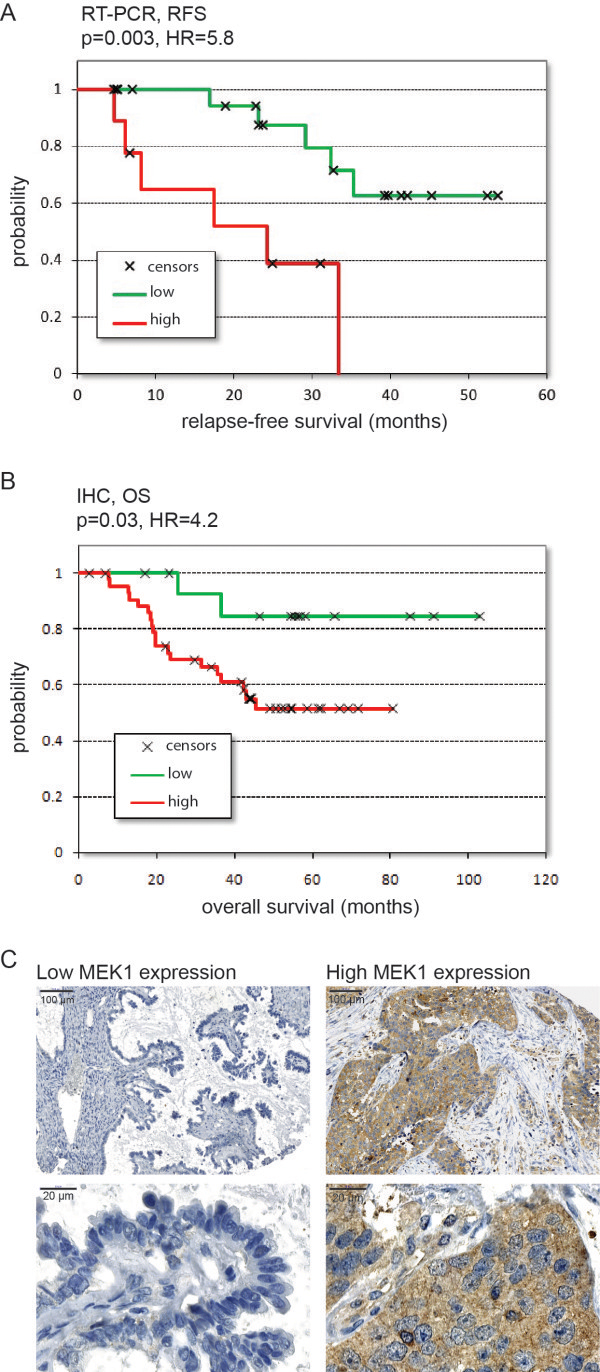
**Correlation between MEK1 expression and survival after platinum treatment in EOC patients.** Expression measured by qRT-PCR: relapse-free survival of 34 patients with low and high MEK1 expressing tumors **(A)**. Expression tested with IHC: overall survival of 59 independent patients with low and high MEK1 expression **(B)**. Representative images of immunohistochemistry, low and high expression of MEK1 at low and high magnifications **(C)**.

All together 44 patient samples were collected at the National Institute of Oncology. 10 patients, not receiving a taxol-carboplatin treatment were excluded. The relative expression values (compared to GAPDH) and the clinical data of the 34 included patients are listed in Additional file 2: Table S2. These patients had a mean relapse-free survival of 25 months. Lower expression of MEK1 (upper quartile vs. remaining samples) significantly correlated with longer relapse-free survival (HR = 5.8, p = 0.003) (Figure 4A).

### Immunohistochemistry

All together samples from 73 independent patients were evaluated. Only patients receiving a platinum-based chemotherapy were included in the IHC evaluation (n = 59). Mean overall survival in these patients was 44.6 months. High staining intensity of MEK1 (upper quartile vs. remaining samples) significantly correlated with worse overall survival in platinum treated patients (HR = 4.2, p = 0.03) (see Figure 4B). The clinical data and detailed results of IHC are listed in Additional file 3: Table S3*.*

### Comparison of MEK1 mediator mechanisms

We utilized the available genomic data to identify the most relevant mechanisms linking carboplatin resistance to MEK1. In this, we computed correlation between MEK1 and ERCC1, the AKT and EMT metagenes *(selection was based on literature search, seeDiscussion)*. The only one of these displaying a significant correlation was the AKT pathway (Spearman correlation coefficient (0.2, p = 2E-12).

## Discussion

The goal of present study was to identify a predictive biomarker of platinum resistance in ovarian cancer. A bottom up approach was set up using an extensive bioinformatic data mining process, in which public transcriptomic and clinical data of more than 1100 ovarian cancer patients was utilized. This number is higher than in any previous study thereby providing a robust foundation for our investigation. Genes showing the highest correlation with clinical response and survival were validated in *in vitro* setting. Finally, the strongest biomarker candidate - MEK1 - was validated in two independent clinical cohorts using qRT-PCR and immunohistochemistry.

The mitogen-activated protein kinase (MAPK) cascade is a key signal transducer of growth factor induced signals and a widely used target of small molecular inhibitors [19, 20]. Within this pathway, MEK1 (MAP2K1) is a MAP kinase kinase impinging on ERK activation, thereby conducting proliferation and anti-apoptotic signals. EGFR, the main activator of MAPK cascade is overexpressed in 70% of ovarian cancers and is associated with worse prognosis, and chemoresistance. Targeting EGFR has a moderate effect in ovarian cancer [21], probably due to collateral escaping mechanisms [22, 23], which could be avoidable by targeting downstream members of the oncogenic pathway. Rahman and colleagues showed that there is correlation between MEK1 amplification (a downstream member of EGFR pathway) and worse progression-free survival in ovarian carcinoma patients [24]. The same association was found in a more recent paper, based on protein profiling data [25]. These are supporting our result that MEK1 overexpression is an independent biomarker of worse survival.

MEK1 inhibitors as targeted therapy agents are already in clinical trials. PD0325901, a selective MEK1 inhibitor - also used in our experiments - was proved to be effective in several preclinical models investigating malignant melanoma and papillary thyroid carcinoma [26, 27] and was already investigated in three clinical trials. Severe musculoskeletal, neurological, and ocular toxicities lead to the termination of a phase I study involving 13 patients with metastatic melanoma, breast or colon cancers [28]. A phase II study investigating the efficacy of PD0325901 in non-small-cell lung cancer was terminated in 2007 due to lack of objective response (unpublished data, clinicaltrials.gov identifier: NCT00174369). Currently, a phase I study is recruiting patients with advanced cancer for a combination trial with two arms: PD-0325901 plus PF-05212384 (an intravenous PI3K/mTOR inhibitor) and PF-05212384 plus irinotecan (clinicaltrials.gov identifier: NCT01347866).

There are several cell-line based studies related to MEK and platinum resistance, although the results are controversial. Some studies show that the platinum induced MEK and ERK activation and overexpression leads to apoptosis [29–32]. Meanwhile others, especially the ones which use ovarian cancer cell lines show the opposite: MEK1 activation leads to platinum resistance [33, 34]. Although these investigations were made in tissue culture, and there is no previous study which associate MEK1 expression with clinical resistance. One of the potential mechanisms linking MAPK pathway to platinum resistance is via a crosstalk with AKT pathway [35]. Overexpression of AKT was associated with chemotherapy resistance [36, 37]. AKT can be activated not only by extracellular growth factor signals, but by activation of DNA-PK (DNA dependent protein kinase) which was described to be overexpressed in platinum resistant high-grade serous ovarian carcinomas [38]. MEK1 can activate the transcription factor ERK, a key activator of proliferation signals [39]. Activation of ERK in cisplatin resistance was shown previously [40] *.* MEK1 activation can cause platinum resistance due to the activation of ERCC1, a well-known molecule in platinum resistance [41]. ERCC1 is a member of the nucleotide exchange repair system, and can induce platinum resistance by removal of platinum adducts from the DNA. Furthermore, MEK1 can also influence platinum resistance by MEK1 induced epithelial-mesenchymal transition (EMT). EMT – a process of epithelial cells losing their epithelial phenotype and transforming to a mesenchymal cell – correlates with higher metastatic activity, more aggressive disease and drug resistance [22]. We computed correlation between MEK1 and these three features (ERCC1, AKT and EMT) in the clinical transcriptomic dataset utilized in our study to rank these mechanisms. We found that only the AKT pathway showed significant correlation with the MEK1 expression.

One of the histological and molecular subtypes of ovarian cancer is low-grade serous (LGS) ovarian carcinoma characterized by BRAF, KRAS, NRAS and ERBB2 mutation, amplification or overexpression [42, 43]. In addition, LGS tumors are highly resistant to chemotherapy [44]. These attributes make LGS tumors a rational candidate for anti-MEK1/2 therapy. In a recent single arm phase two clinical trial in patients with recurrent LGS ovarian cancer, the MEK1/2 inhibitor selumetinib achieved response only in 15% of patients according to the RECIST criteria [45]. In our study, massive cell death was observed after inhibition of MEK1 in combination with even a very low dose of carboplatin. Instead of inhibiting MEK1 using a single agent, our results propose to use it in combination with carboplatin as a sensitizing agent in high grade tumors.

## Conclusion

Since the 1970’s, significant improvement was achieved in the treatment of ovarian cancer patients and the five-year overall survival increased by 25 percent [46]. However, current platinum-based treatment protocols are still far from optimum, and we can only improve outcome by identifying and stimulating more robust targets. In our study, by employing *in silico* and *in vitro* analysis coupled with independent validation in clinical cohorts, we identified *MEK1* as a promising prognostic biomarker candidate correlated to response to platinum based chemotherapy in ovarian cancer. Furthermore, we could also restrain platinum resistance by targeting MEK1. Our results could allow the utilization of a more targeted therapy and the development of more efficient anticancer therapies for ovarian cancer.

## Electronic supplementary material

Additional file 1: Table S1: Used carboplatin and PD0325901 concentration ranges in the in vitro experiments. (XLSX 9 KB)

Additional file 2: Table S2: Clinical parameters and qRT-PCR based expression of the validation group. Patient ID, histology of the tumor, grade, stage, surgical result, chemotherapy (TXL-CRB means taxol-carboplatin) and survival data, together with the relative expression of the investigated genes. (XLSX 14 KB)

Additional file 3: Table S3: Clinical parameters and IHC based expression of the validation group. Patient ID, chemotherapy and survival data, together with the staining signal of MEK1. (XLSX 12 KB)
